# Safety and reliability of telesurgery in China: a multicenter, single-arm, phase I clinical trial

**DOI:** 10.1097/JS9.0000000000002792

**Published:** 2025-07-02

**Authors:** Sheng Tai, Ye Wang, Sunyi Ye, Tao Xu, Gen Cheng, Cheng Yang, Hongzhi Wang, Haibing Xiao, Qingbo Huang, Shuo Wang, Xiangping Zhang, Jianle Mao, Nan Qiao, Zongyao Hao, Dan Xia, Wanhai Xu, Xu Zhang, Chaozhao Liang

**Affiliations:** a Department of Urology, The First Affiliated Hospital of Anhui Medical University, Hefei, China; b Department of Urology, Chinese PLA General Hospital, Beijing, China; c Department of Urology, The First Affiliated Hospital, School of Medicine, Zhejiang University, Hangzhou, China; d Department of Urology, Harbin Medical University Cancer Hospital, Harbin, China; e Department of Clinical Coordination, The Shenzhen Edge Medical Co., Ltd., Shenzhen, China.

**Keywords:** China, safety and reliability, telesurgery

## Abstract

**Background:**

The development and implementation of telesurgery are of significant strategic importance for the future expansion and evolution of the surgical robotics industry. Telesurgery has been demonstrated to improve access to advanced medical care but also enhances the overall healthcare delivery in these underserved regions. Here, we presented a telesurgery system that optimized surgical signals via a telesurgery host using real-time signal processing and multi-source signal interaction systems. Additionally, a telesurgery network solution was developed to ensure seamless network switching.

**Materials and methods:**

We present the results of the first multi-center human study of robot-assisted laparoscopic telesurgery. Specifically, telesurgeries were performed on 18 patients across four hospitals in China utilizing EDGE MP 1000 tele-surgical systems. The distances between each pair of cities ranged from 450 km to 2200 km.

**Results:**

The telesurgery success rate was 100%. The mean real-time round-trip network latency, network jitter, video encoding/decoding latency, code rate, and video display latency were 38.38 ± 13.25 ms, 0.39 ± 0.29 ms, 20.02 ± 0.06 ms, and 12.77 ± 0.57 Mbps, 21.35 ± 3.42 ms, respectively. There was no frame loss during all telesurgeries.

**Conclusion:**

Our results indicated that telesurgery using 5G and dedicated line technology was a safe and feasible treatment option for patients with urologic oncology.

## Introduction

The advent of telesurgery represents a significant milestone in the evolution of surgical practices, driven by the need to provide high-quality surgical care to patients in remote or underserved regions by overcoming geographical barriers. Initially conceptualized during wartime to enable surgeons to perform complex procedures on trauma patients located far from medical facilities, the practical application of telesurgery was hindered for many years by the limitations of early robotic systems and telecommunication technologies^[1–9]^.

**HIGHLIGHTS**

- The 18 patients from four hospitals successfully underwent telesurgery.
- The mean real-time round-trip network latency was 38.38 ± 13.25 ms.
- The video encoding/decoding latency was 20.02 ± 0.06 ms, and network jitter was 0.39 ± 0.29 ms.
- The code rate was 12.77 ± 0.57 mbps, and video display latency was 21.35 ± 3.42 ms.

In recent years, disparities in healthcare resource distribution and the challenges posed by the global COVID-19 pandemic have amplified the need for remote surgical solutions^[10–13]^. Patients in remote or underdeveloped areas often lack access to timely and specialized surgical care, creating an urgent demand for innovative approaches to bridge this gap. Modern advancements in telecommunication systems, high-speed networks, and sophisticated robotic surgical systems have now paved the way for the practical implementation of telesurgery^[14–20]^.

The world’s first telesurgery was performed in New York in 2001 using the ZEUS robotic system^[21]^. In 2005, Sterbis *et al* performed a remote robot-assisted laparoscopic right nephrectomy via a commercial wired network^[22]^. Recently, Chinese medical teams have been actively exploring telesurgery using domestically produced robotic systems^[23]^. Liu Rong’s team completed a 5G remote experimental liver wedge resection in pigs, preliminarily validating the feasibility of 5G-based domestic telesurgery technology^[24]^. Yang *et al* used the MicroHand surgical robotic system to perform a remote robot-assisted laparoscopic radical cystectomy through a 5G network^[25]^. Fan and colleagues carried out dual-console remote laparoscopic surgeries with the KANDUO system using both 5G and wired networks^[26]^. Zhang Xu’s team also successfully conducted a series of urologic telesurgeries with a dedicated line over 3000 km^[27]^.

In this study, we proposed a novel telesurgery system that preprocessed multiple signals through a telesurgery host. This system ensured seamless network switching via a telesurgery network solution, facilitating successful surgical procedures. Additionally, we designed the world’s first multicenter, long-distance telesurgery study to assess the safety and feasibility of robot-assisted laparoscopic telesurgery for partial nephrectomy and prostatectomy across four cities, in compliance with the TITAN Guidelines 2025^[28]^.

## Methods

### Study design

This multicenter, single-arm, phase I clinical trial was conducted in four different regions in China. The study aimed to investigate the reliability and stability of telesurgery. The study was approved by the ethics committees of all participating institutions and registered on ClinicalTrials.gov and was performed in accordance with the 2013 Declaration of Helsinki. Ethical principles. All patients were thoroughly informed by experienced urologists and voluntarily signed informed consent forms prior to surgery.

### Telesurgery system

**Figure 1.**
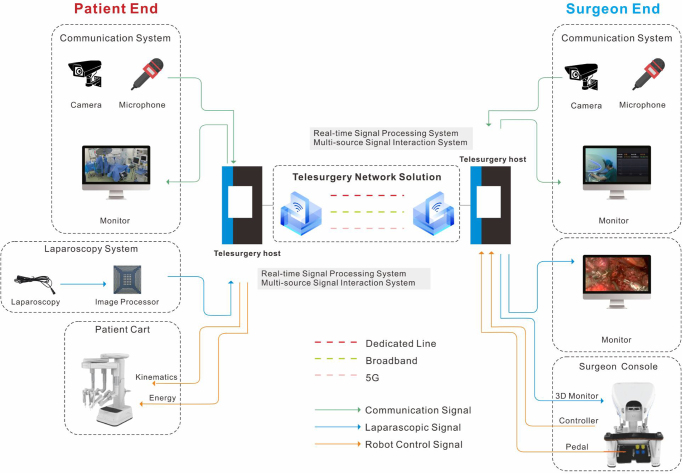
The telesurgery system in this study. The telesurgery system comprises a complete surgical robotic system with a console and robotic arms, alongside a telecommunications system and a teleconferencing system. Signals aggregated to the telesurgery host were optimized using real-time signal processing and multi-source signal interaction systems. The telesurgery network solution ensured seamless network switching, supporting the coexistence of three networks and multi-point interconnectivity.

The telesurgery system consisted of four independent subsystems: a surgeon console (at the master arm side) and a bedside operating system (at the slave arm side), a teleconference system, and a telecommunications system (Fig. 1). The MP1000 Surgical Robotic System (Shenzhen Edge Medical Co., Ltd., Shenzhen, China) was used in this study^[29]^ (Supplementary Digital Content, Fig. 1, available at: http://links.lww.com/JS9/E577).

Signals from the four subsystems were aggregated to the telesurgery host, in which surgical signals were optimized using real-time signal processing and multi-source signal interaction systems. Real-time signal processing methods included ultra-low latency codec optimization, full with 2K/4K 3D video resolution compatibility (Supplementary Digital Content, Videos 1 and 2, available at: http://links.lww.com/JS9/E581 and http://links.lww.com/JS9/E582), adaptive bitrate and resolution adjustment, forward error correction, network jitter buffering, and region of interest (ROI)-adaptive network adjustment. Multi-source signal interaction optimization techniques encompassed ultra-low bandwidth control algorithms, audio-video transmission control algorithms, intelligent jitter buffer adjustment, weak network resistance, and audio noise reduction.

Additionally, the telecommunications network utilized three types of connections: a dedicated line, a commercial broadband, and a 5G network. A telesurgery network solution was developed to support the coexistence of the three networks and enable seamless switching between them. This solution also supports hot standby for network links and multi-point interconnectivity (Fig. 1).

One telesurgery network was constructed between two city pairs in China (Fig. 3). The mean real-time round-trip network latency, network jitter, video encoding/decoding latency, code rate, and video http://links.lww.com/JS9/E582 display latency were all recorded in the study.

### Inclusion and exclusion criteria

The inclusion criteria were as follows: (1) age was 18–80 years; (2) BMI was 18–30 kg/m^2^; (3) indication for partial nephrectomy and radical prostatectomy; and (4) patients volunteered to join the study and signed the consent. The exclusion criteria were as follows: (1) patients with severe cardiovascular disease with New York cardiac class III–IV or pulmonary insufficiency; (2) patients with epilepsy or psychosis; (3) female patients during the gestation or lactation periods; (4) patients with severe allergies, or addiction to alcohol and drugs; (5) patients with severe cardiac, pulmonary, renal, and hepatic insufficiency; (6) patients participated in any other clinical trial; and (7) other conditions.

### Sample size calculation

In this single-arm clinical trial, the sample size was determined to be 18 patients. This sample size was calculated based on the study’s primary objective, which was to evaluate the feasibility and safety of the telesurgery system. The calculation considered a 95% confidence level, a 5% margin of error, and an expected 90% success rate. Given the exploratory nature of this pilot study, the chosen sample size was sufficient to provide preliminary data on the system’s performance and potential clinical benefits.

### Telesurgery process

Local urologists from different centers performed the surgeries, and all medical staff received adequate training. Surgeons were required to complete at least 500 robot-assisted laparoscopic surgeries. To reduce the heterogeneity, all surgeons received instructions from the supervisor. In the event of a malfunction, the local surgeon can immediately and seamlessly take control of the robot through the local surgeon console, ensuring surgical safety.

### Primary and secondary parameters

In the study, primary parameter was the telesurgery success ratio and secondary parameters for the evaluation of telesurgery included operative time, warm ischemia time, prostate specific antigen (PSA) level after surgery, blood loss, positive margin rate, postoperative hospitalization days, adjacent organ injuries, postoperative complications, patients’ anxiety, depression scores and pain levels, and the surgeon’s subjective evaluation, including the NASA-TLX (National Aeronautics and Space Administration Task Load Index) scores (Supplementary Digital Content, Table 1, available at: http://links.lww.com/JS9/E578) and a telesurgery-relating index (Supplementary Digital Content, Table 2, available at: http://links.lww.com/JS9/E579).

**Table 1:** Patients’ demographics and clinical outcomes

	Partial nephrectomy	Prostatectomy
Patient number, *n* (%)	7 (38.9%)	11 (61.1%)
Median age (mean ± SD)	57.1 ± 6.6	68.3 ± 7.5
Gender, *n* (%)		
Male	4 (42.8%)	11 (100%)
Female	3 (57.2%)	
BMI (mean ± SD)	24.06 ± 3.05	23.21 ± 2.54
TNM, *n* (%)	T1aN0M0, 4 (57.2%)	T2N0M0, 9 (81.8%)
	T1bN0M0, 3 (42.8%)	T3aN0M0, 1 (9.1%)
		T3bN0M0, 1 (9.1%)
Operative time (mean ± SD), min	127.4 ± 37.9	167.8 ± 30.0
tPSA (mean ± SD), ng/mL		13.83 ± 7.73
Gleason score (median, IQR)		7 (6–7)
Renal score (median, IQR)	8 (5–8)	
Warm ischemia time (mean ± SD), min	19.3 ± 9.6	
Blood loss (median, IQR), mL	20 (20–100)	50 (30–50)
Tumor size (mean ± SD), cm	3.03 ± 1.26	4.83 ± 0.76
Positive margin rate, *n* (%)	0 (0)	0 (0)
Postoperative hospitalization days (median, IQR)	5 (5–6)	4 (4–5)
Adjacent organ injures, *n* (%)	0 (0)	0 (0)
Postoperative complications, *n* (%)	2 (28.6%)	2 (18.2%)
Clavien–Dindo grade 1	1 (14.3%)	1 (9.1%)
Clavien–Dindo grade 2	1 (14.3%)	1 (9.1%)
Clavien–Dindo grade 3	0 (0)	0 (0)
Renal tumor recurrence or metastasis, *n*(%)	0 (0)	
At 10 month after surgery		
Prostate-specific antigen level, *n* (%)		
At 1 month after surgery		
>0.1 ng/mL		2 (18.2%)
0.02–0.1 ng/mL		4 (36.4%)
<0.02 ng/mL		5 (45.4%)
Prostate-specific antigen level, *n* (%)		
At 10 month after surgery		
>0.1 ng/mL		0 (0)
0.02–0.1 ng/mL		6 (54.5%)
<0.02 ng/mL		5 (45.5%)
SDS, (median, IQR)	42 (37–48)	29 (23–32)
SAS, (median, IQR)	47 (40–51)	35 (30–37)
Pain, (median, IQR)	2 (2–3)	1 (0–3)

BMI, body mass index; IQR, interquartile range; SAS, Self-Rating Anxiety Scale; SD, standard deviation; SDS, Self-Rating Depression Scale.

**Table 2:** Number of patients, distance, and parameters of telesurgery network of five city pairs: Hefei–Beijing, Hefei–Hangzhou, Hefei–Harbin, Beijing–Hangzhou, and Harbin–Hangzhou

	Number of patients	Distance (km)	The real-time round-trip network latency (ms) (mean ± SD)	Network jitter (ms) (mean ± SD)	Video encoding/decoding latency (ms) (mean ± SD)	Frame loss	Code rate (mbps) (mean ± SD)	Video display latency (ms) (mean ± SD)
Hefei–Beijing	4	1000 km	26.41 ± 3.15	0.38 ± 0.28	20.02 ± 0.06	0	12.74 ± 0.5687	25.52 ± 5.005
Hefei–Hangzhou	10	450 km	16.59 ± 0.80	0.38 ± 0.28	20.02 ± 0.07	0	12.80 ± 0.6191	20.02 ± 0.379
Hefei–Harbin	1	2000 km	50.68 ± 0.22	0.36 ± 0.28	20.02 ± 0.07	0	12.76 ± 0.5454	20.05 ± 0.574
Beijing–Hangzhou	1	1300 km	26.33 ± 0.15	0.42 ± 0.28	20.02 ± 0.07	0	12.81 ± 0.2230	20.03 ± 0.572
Harbin–Hangzhou	2	2200 km	53.31 ± 0.31	0.44 ± 0.30	20.02 ± 0.07	0	12.76 ± 0.4315	20.03 ± 0.480

SD, standard deviation.

### Statistical analyses

Statistical analyses were performed using standard methods. The normality of the data was assessed using the Shapiro–Wilk test. Descriptive statistics were calculated to summarize patient characteristics and outcomes. These statistics included means and standard deviations for continuous variables and frequencies and percentages for categorical variables. For normally distributed data, comparisons between groups were conducted using an independent-samples *t*-test. A *P*-value of less than 0.05 was considered statistically significant. All statistical analyses were performed using GraphPad Prism 6.0.

## Results

### Patients’ recruitment

**Figure 2.**
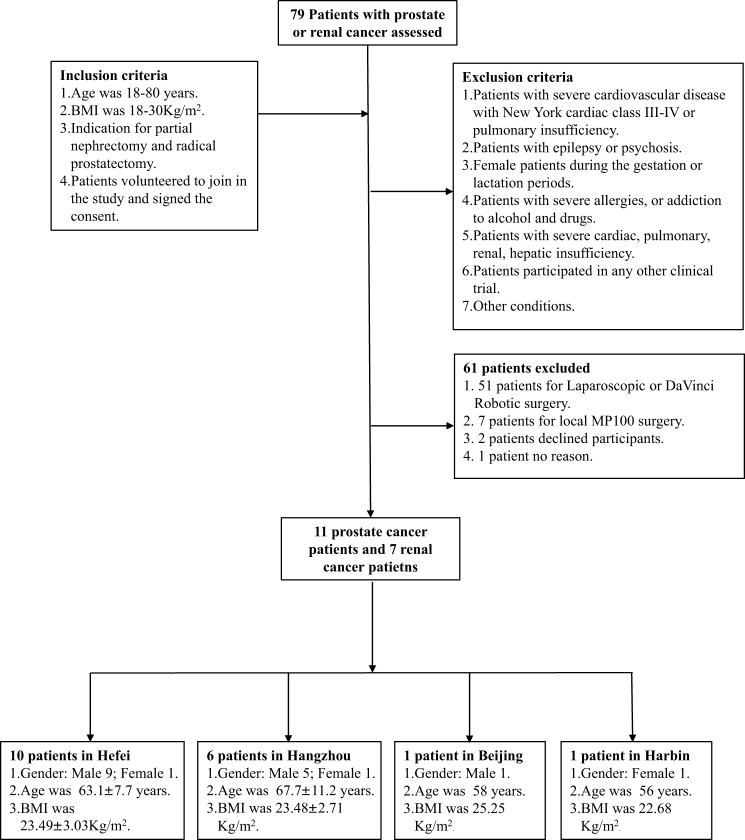
The inclusion, exclusion, and screening process of patients is detailed.

**Figure 3.**
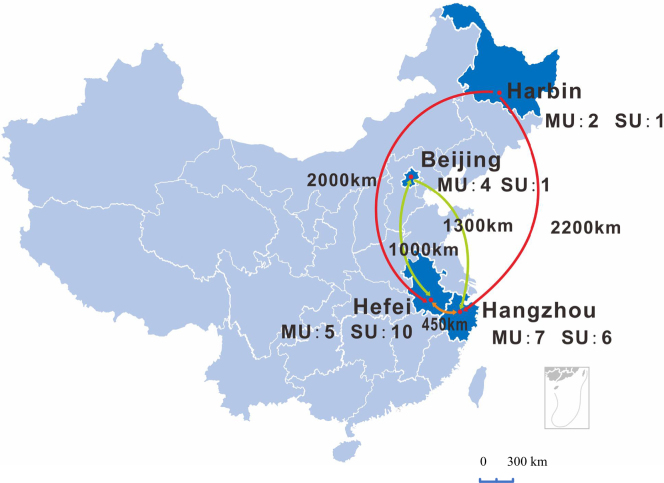
The location of the four cities in this study. The locations of the four cities in China: Harbin to Hangzhou encompasses the farthest distance, spanning up to 2200 km, and the number of surgeon side (master unit, MU) and patient side (salve unit, SU) in each city.

A total of 18 patients from four hospitals were recruited for this study, undergoing either robot-assisted laparoscopic partial nephrectomy (7 patients, 38.9%) or robot-assisted laparoscopic radical prostatectomy (11 patients, 61.1%). Figure 2 details the inclusion, exclusion, and screening process of patients. Figure 3 shows the number of surgeon sides (master unit, MU) and patient sides (slave unit, SU) in each city. In Hefei, the number of MUs and SUs was 5 and 10, respectively. In Hangzhou, the number of MUs and SUs was seven and six, respectively. In Beijing, the number of MUs and SUs was 4 and 1, respectively. In Harbin, the number of MUs and SUs was 2 and 1, respectively (Fig. 3).

### Patients’ demographics and clinical outcomes

Patients’ demographics, preoperative and postoperative conditions, were assessed, as detailed in Table 1. All 18 patients successfully underwent telesurgery. The patients’ demographics and peri-operative details are shown in Table 1. Adjacent organ injuries were all negative, and all surgical margins were pathologically negative. For the seven patients who underwent partial nephrectomy, the operative time was 127.4 ± 37.9 min, renal score was 8 [interquartile range (IQR), 5–8], blood loss was 20 (IQR, 20–100) mL, postoperative hospitalization was 5 (IQR, 5–6) days, and postoperative complications occurred in two patients (28.6%), mainly fever and infection. For the 11 patients who underwent prostatectomy, the operative time was 167.8 ± 30.0 min, blood loss was 50 (IQR, 30–50) mL, postoperative hospitalization was 4 (IQR, 4–5) days, and postoperative complications occurred in two patients (18.2%), primarily fever and pain. The tPSA was 13.83 ± 7.73 ng/mL. One month postoperatively, PSA levels were >0.1 ng/mL in two patients (18.2%), 0.02–0.1 ng/mL in four patients (36.4%), and <0.02 ng/mL in five patients (45.4%). Ten months postoperatively, PSA levels were 0.02–0.1 ng/mL in six patients (54.5%), and <0.02 ng/mL in five patients (45.5%). There was no renal tumor recurrence or metastasis at the 10-month follow-up. Additionally, postoperative pain, anxiety, and depression were assessed. For partial nephrectomy patients, pain scores were 2 (IQR, 2–3), Self-Rating Depression Scale (SDS) was 42 (IQR, 37–48), and Self-Rating Anxiety Scale (SAS) was 47 (IQR, 40–51). For prostatectomy patients, pain scores were 1 (range 0–3), SDS was 29 (IQR, 23–32), and SAS was 35 (IQR, 30–37). Results indicated mild pain, depression, and anxiety in patients. Hemoglobin and creatinine from the preoperative and postoperative two follow-up visits were shown in the Supplementary Digital Content, Table 3, available at: http://links.lww.com/JS9/E580.

### Telesurgery parameters

**Figure 4.**
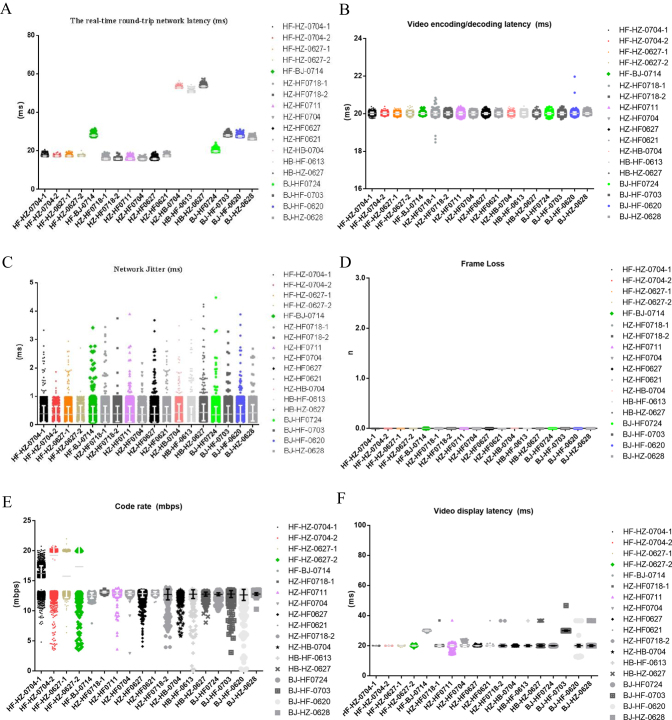
The network parameters of telesurgery. (A) The average real-time round-trip network latency between each city pair ranged from 15.81 to 53.49 ms. (B) The video encoding/decoding latency remained stable at approximately 20 ms. (C) The network jitter was within a narrow range of 0.36–0.44 ms. (D) No frame loss occurred during the surgeries. (E) The average code rate ranged from 15.81 to 53.49 ms, with minimal fluctuation. (F) The average video display latency ranged from 20.02 to 25.52.

Among all telesurgeries, the mean real-time round-trip network latency was 38.38 ± 13.25 ms (Fig. 4A), the video encoding/decoding latency was 20.02 ± 0.06 ms (Fig. 4B), and the network jitter was 0.39 ± 0.29 ms (Fig. 4C). There was no image frame loss (Fig. 4D). The code rate was 12.77 ± 0.57 Mbps (Fig. 4E), and the video display latency was 21.35 ± 3.42 ms (Fig. 4F). Surgeries were performed across five city pairs: Hefei–Beijing (1000 km, 4 surgeries), Hefei–Hangzhou (450 km, 10 surgeries), Hefei–Harbin (2000 km, 1 surgery), Beijing–Hangzhou (1300 km, 1 surgery), and Harbin–Hangzhou (2200 km, 2 surgeries). Round-trip network latency increased with distance. The shortest delay was 16.59 ± 0.80 ms between Hefei–Hangzhou, and the longest delay was 53.31 ± 0.31 ms between Harbin–Hangzhou (Table 2). The parameters, including network jitter, video encoding/decoding latency, frame loss, code rate, and video display latency remained stable across different city pairs and did not fluctuate with increasing geographical distance (Table 2). The real-time signal processing and multi-source signal interaction systems combined with the telesurgery network solution enabled intelligent optimization of data signals at multiple stages: before, during, and after transmission. This ensured stable and high-quality data transmission throughout the process.

### The NASA-TLX assessment and subjective evaluation of telesurgery

**Figure 5.**
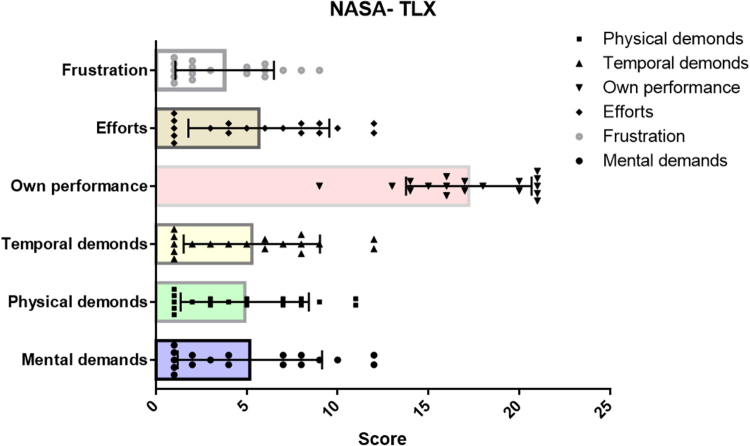
The NASA-TLX assessment. The postoperative NASA-TLX assessment results of the primary surgeon, including mental demands, physical demands, temporal demands, own performance, efforts, and frustration.

**Figure 6.**
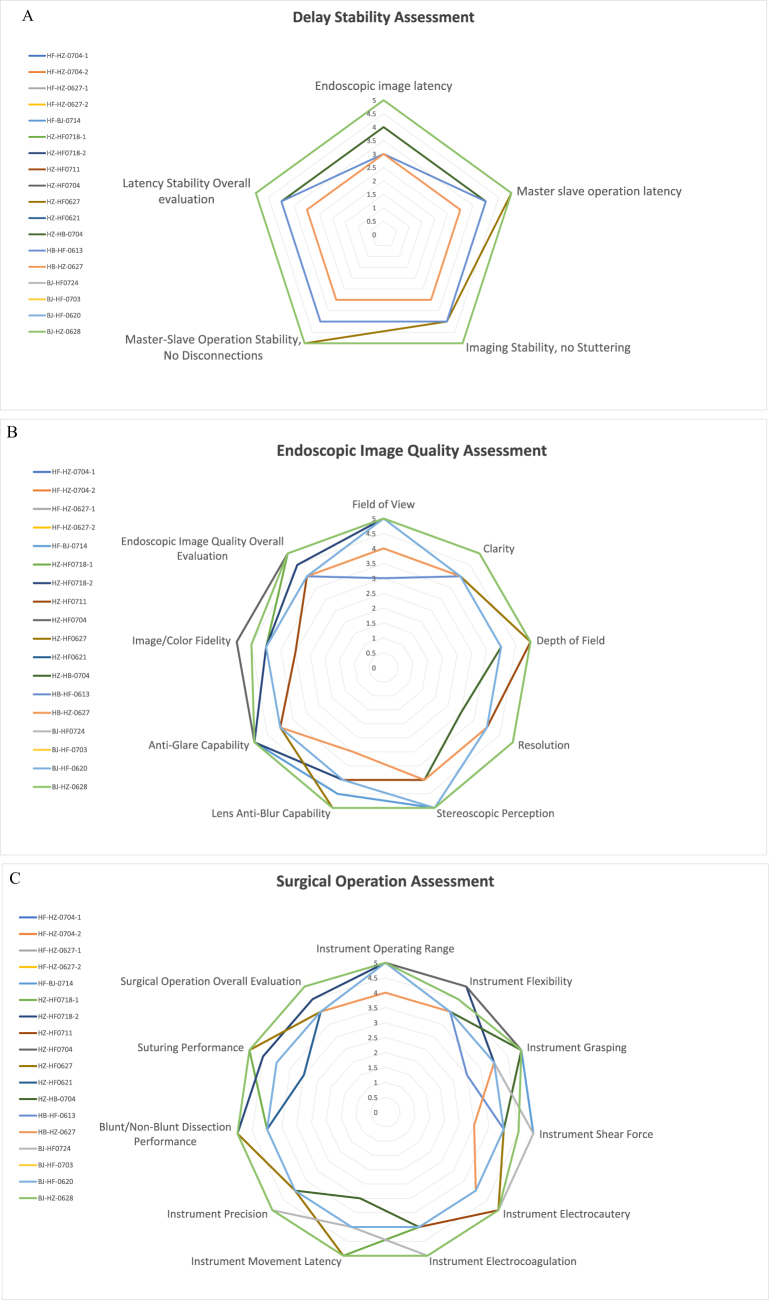
Surgeon’s subjective evaluation of telesurgery. The questionnaire used 5, 9, and 11 indicators to assess delay stability (A), endoscopic image quality (B), and surgical operation (C). Overall workload assessment scores were 4 or 5 in 96% of all items. Score 1: completely impossible; Score 2: somewhat possible but almost impossible; Score 3: uncomfortable but possible; Score 4: somewhat of a discomfort but possible; and Score 5: possible.

The NASA-TLX was utilized to assess the workload of physicians during surgical procedures. The assessment included six subscales, with the following results: mental demand was 5.2 ± 4.0, physical demand was 4.9 ± 3.5, temporal demand was 5.3 ± 3.8, performance was 17.2 ± 3.4, effort was 5.7 ± 3.9, and frustration was 3.8 ± 2.7. The cumulative score across all subscales was 42.0 ± 15.2 (Fig. 5). These results suggest that, the surgical procedures did not impose a significant workload on the physicians, as indicated by the relatively moderate scores across most subscales and a total score that reflected manageable task demands. Surgeons evaluated latency stability using five indicators: endoscopic image latency, master-slave operation latency, imaging stability (no stuttering), master-slave operation stability (no disconnections), and overall latency stability (Fig. 6A). Scores of 3 or less were given in ≤2/18 (11.1%) cases. Endoscopic image quality was assessed using nine indicators: field of view, clarity, depth of field, resolution, stereoscopic perception, lens anti-blur capability, anti-glare capability, image/color fidelity, and overall endoscopic image quality (Fig. 6B), with scores of 3 or less in ≤4/18 (22.2%) cases. Surgical operation was rated using 11 indicators: instrument operating range, flexibility, grasping, shear force, electrocautery, electrocoagulation, movement latency, precision, blunt/non-blunt dissection performance, suturing performance, and overall surgical operation (Fig. 6C), with scores of ≤3 in ≤3/18 (16.7%) cases. Overall workload assessment scores were 4 (somewhat discomfort but possible) or 5 (possible) in 96% of all items, indicating that surgeons subjectively rated the surgical process as relatively smooth.

## Discussion

In this study, we utilized the Edge MP1000 surgical robot to develop a telesurgery system composed of four components: a surgeon console, a bedside operating system, a teleconference system, and a telecommunications system. We optimized surgical signals using real-time signal processing and multi-source signal interaction systems. Additionally, we developed a telesurgery network solution to ensure seamless network switching. We conducted a multicenter one-arm, clinical study to validate the feasibility and efficacy of the telesurgical system. The telesurgery success rate was 100%, demonstrating that the telesurgery system is safe and effective.

During the study, NASA-TLX scores ranged from 42.0 ± 15.2 (Fig. 5). Overall subjective assessment scores were 4 or 5 for 96% of items (Fig. 6), indicating that telesurgery did not increase surgical workload. Surgeons subjectively rated the surgical process as relatively smooth. The mean values for real-time round-trip network latency, network jitter, video encoding/decoding latency, code rate, and video display latency were 38.38 ± 13.25 ms, 0.39 ± 0.29 ms, 20.02 ± 0.06 ms, 12.77 ± 0.57 Mbps, and 21.35 ± 3.42 ms, respectively. There was no frame loss during all telesurgeries. Previous research had suggested that a latency of less than 200 ms was considered ideal for surgical conditions, as it was imperceptible to the surgeon and did not impact surgical performance^[30–32]^. The total latency in our study was well below this threshold, aligning with surgeons’ subjective experiences and ensuring the smooth execution of the surgeries.

Due to the involvement of multiple signal transmissions during surgery, we used a telesurgery host to pre-process signals with real-time signal processing and multi-source signal interaction systems. These systems handled both real-time and multi-source signals, incorporating ultra-low latency codec optimization, full compatibility with 2K/4K 3D video resolution, adaptive bitrate and resolution adjustment, forward error correction, network jitter buffering, and ROI-adaptive network adjustment. Additionally, ultra-low bandwidth control algorithms, audio-video transmission control algorithms, intelligent jitter buffer adjustment, weak network resistance, and audio noise reduction were employed.

Given the significant distances between Beijing, Hangzhou, Hefei, and Harbin, this study utilized China Unicom’s AAA-grade Optical Transport Network line with 60 Mbps bandwidth, along with commercial broadband and 5G networks for data transmission. A telesurgery network solution was developed to ensure seamless network switching, supporting the coexistence of three networks, network switching, hot standby for network links, and multi-point interconnectivity (Fig. 1). This study also had limitations, including a small sample size, single-arm design, and potential influences from patient awareness and varying surgeon experiences on hospital duration. Further research involving larger-scale trials and diverse surgical procedures across different specialties is necessary to validate the application of telesurgery in more complex surgeries.

In conclusion, this multicenter, single-arm clinical trial demonstrated the safety and feasibility of telesurgery using the Edge MP1000 system. This approach may help address the uneven distribution of medical resources and support remote medical rescues during disasters or wartime.

## Data Availability

Data may be provided if it is necessary.

## Research registration unique identifying number (UIN)

ClinicalTrials.gov (ChiCTR2400085386).
